# Dual analysis of postural control in middle-aged and elderly patients with cervicogenic dizziness: Dynamic and static balance perspectives

**DOI:** 10.3389/fbioe.2025.1622648

**Published:** 2025-07-22

**Authors:** Wei Luo, Yu Min, Peishun Chen, Hao Li, Zhiyong Long, Ju Sun, Tao Zhong

**Affiliations:** Department of Rehabilitation Medicine, The Affiliated Panyu Central Hospital, Guangzhou Medical University, Guangzhou, Guangdong, China

**Keywords:** middle-aged and elderly, cervicogenic dizziness, postural control, static balance, limits of stability

## Abstract

**Objectives:**

This study aimed to comprehensively analyze the postural control characteristics of middle-aged and elderly patients with cervicogenic dizziness from both dynamic and static balance perspectives.

**Methods:**

A cross-sectional study was conducted involving 20 patients with cervicogenic dizziness (dizziness group) and 20 healthy individuals (health group). Using the Prokin Balance Instrument, we conducted static balance and limits of stability tests on both groups. Key metrics such as average speed of sway, standard deviation of sway, average center of pressure, Romberg’s ratio, and limits of stability values were recorded.

**Results:**

With the exception of the standard deviation of mediolateral sway in the healthy group, the values of static balance indices were higher in the eyes-closed condition compared to the eyes-open condition for both groups (|Z| > 2.068, P < 0.05). Except for the average speed of mediolateral sway in both the eyes-open and eyes-closed conditions and the length of body sway in the eyes-open condition, the remaining static balance index values were higher in the dizziness group than in the healthy group (|Z| > 2.077, P < 0.05). Compared to the healthy group, the Romberg ratio was significantly higher in the dizziness group, while the values of the limits of stability were significantly lower (P < 0.05). Furthermore, the average center of pressure along the X and Y-axes exhibited a dispersed distribution pattern away from the axis in the dizziness group, in contrast to the healthy group, which demonstrated a concentrated distribution pattern close to the axis.

**Conclusion:**

Middle-aged and elderly patients with cervicogenic dizziness demonstrate postural control abnormalities, including decreased static balance, reduced limits of stability, increased center of gravity sway, reliance on visual compensation for postural control, and an elevated risk of falls.

## 1 Introduction

Cervicogenic dizziness is a prevalent clinical condition characterized by dizziness and balance disorders resulting from neck issues. As a significant component of dizziness-related disorders, cervicogenic dizziness accounts for approximately 89% of all dizziness cases (Takahashi, 2018; Chu et al., 2023). Epidemiological studies indicate a prevalence of 10% in adults, with a higher incidence in the elderly population, affecting about 30% of this group (Yao et al., 2020; Li et al., 2022). Retrospective studies in otorhinolaryngology report non-traumatic cervicogenic dizziness rates ranging from 5.42% to 7.5% (Polaczkiewicz and Olszewski, 2019; Ardiç et al., 2006). A prospective multicenter study further corroborated a prevalence of 6.4% (Lüscher et al., 2014). These data suggest a notable prevalence of cervicogenic dizziness among dizziness patients, although exact figures may vary based on population and study methodology. Importantly, with the increasing use of electronic devices and lifestyle changes, the incidence of cervicogenic dizziness is rising annually, and the age of onset is decreasing (Takahashi, 2018; Tardov et al., 2022). Typical symptoms include dizziness, neck pain, limited mobility, and abnormal postural control (Micarelli et al., 2021; Végh et al., 2019), with severe cases potentially leading to complications such as fractures and stroke (Xie et al., 2020).

Among these symptoms, decreased postural stability is considered the most frequent and consistent clinical feature of cervicogenic dizziness (Knapstad et al., 2019). The pathogenesis of these postural abnormalities remains unclear, though several hypotheses have been proposed, including vascular compression, sympathetic nerve stimulation, cervical proprioceptive disturbances, and migraine-related mechanisms (Végh et al., 2019). The cervical proprioceptive disorder hypothesis is the most widely accepted (Végh et al., 2019). Given that balance is primarily regulated by vision, proprioception, and vestibular sensation, any abnormalities in proprioception can disrupt balance control (Cao et al., 2021). Therefore, characterizing postural control in patients with cervicogenic dizziness is crucial for understanding its pathogenesis and developing targeted therapeutic strategies.

Previous studies, such as the one by Micarelli et al., utilized the postural picture test to evaluate the balance function of patients with cervicogenic dizziness and confirmed that these patients exhibited significant increases in classic postural picture parameters (such as area and length) (Micarelli et al., 2021). However, this study relied on a single balance parameter and lacked high-sensitivity balance measures such as center of gravity sway frequency, amplitude, and limits of stability, thus offering limited insight into balance characteristics. Additionally, research by Micarelli and colleagues using the Dizziness Disorder Scale revealed higher balance scale scores in cervicogenic dizziness patients compared to healthy individuals, reflecting not only limited balance function but also psychological factors like depression and fear (Micarelli et al., 2020). However, this study lacked dynamic balance assessment and employed a highly subjective balance scale, limiting its objectivity. Despite the significant impact of postural control deficits on the quality of life in cervicogenic dizziness patients, there remains a paucity of studies characterizing their balance function. Most clinical studies rely on scales like Berg and Tinetti, or single balance parameters, which are cumbersome and limited in accuracy and objectivity (Taghavi Azar Sharabiani et al., 2024; Chen et al., 2024; Rivolta et al., 2019).

Recently, balance testers have emerged as a preferred tool for balance function research due to their objectivity, accuracy, and ease of use. These devices quantitatively assess static and dynamic balance abilities and identify the degree, type, or cause of balance impairments (Lin et al., 2020). The Prokin Balance tester has been extensively utilized in research across a variety of diseases. For instance, Zhang et al. engaged 20 healthy volunteers to participate in a balance test aimed at investigating postural control in patients with chronic low back pain. The findings revealed that the Prokin Balance tester exhibited excellent intra-rater reliability (Zhang et al., 2020). In a separate study examining dynamic and static balance in stroke patients, researchers compared the Prokin Balance tester with traditional tools like the widely used Berg Balance Scale. The results demonstrated that the Prokin Balance tester offers superior effectiveness and advantages in posture assessment (Lin et al., 2020). Furthermore, in studies exploring postural control in conditions such as acromegaly, migraine, and stroke, the Prokin Balance tester effectively differentiated balance disparities between patients and healthy individuals (Haliloglu et al., 2019; Dumanlidağ and Milanlioğlu, 2021; Lin et al., 2020).

In light of this, we employed the highly reliable and efficient Prokin Balance Instrument (TECNOBODY, Italy, Model 252) to accurately measure and analyze postural control in cervicogenic dizziness patients (Meier et al., 2016). The aim of this study is to elucidate the postural control characteristics of cervicogenic dizziness patients, providing a new perspective and scientific basis for the assessment, diagnosis, and treatment of cervicogenic dizziness, ultimately improving clinical treatment effectiveness and patient quality of life.

We hypothesize that both healthy individuals and patients with cervicogenic dizziness will exhibit higher static balance index values with eyes-closed compared to eyes-open. However, the disparity between these conditions is expected to be significantly more pronounced in patients with cervicogenic dizziness than in the healthy population, as indicated by higher Romberg ratios (the ratio of the eyes-closed condition to the eyes-open condition). Furthermore, patients with cervicogenic dizziness are anticipated to demonstrate markedly reduced limits of stability, greater oscillation amplitude of the center of plantar pressure, and an increased risk of falls compared to healthy subjects.

## 2 Materials and methods

### 2.1 Participants

The sample size estimation for this study was conducted with a significance level of 0.05, a power of 0.8, and an effect size of 0.9, based on insights from prior research (Yahia et al., 2009). Consequently, the study enrolled 20 middle-aged and elderly patients diagnosed with cervicogenic dizziness by a surgeon (dizziness group) at the Affiliated Panyu Central Hospital of Guangzhou Medical University. Concurrently, 20 healthy individuals were recruited as the control group during the same timeframe.

Inclusion Criteria: Participants were selected based on the following criteria (Lin et al., 2020): (a) aged between 46 and 70 years, with stable vital signs and no cognitive impairments; (b) experiencing recurrent episodes of dizziness, neck pain, and restricted mobility; (c) symptoms that are exacerbated by changes in body position or neck movement, which may be persistent or intermittent; (d) imaging studies revealing altered cervical spine curvature, osteophytes at the intervertebral joints, vertebral instability, atlantoaxial subluxation, or disc herniation.

Exclusion Criteria: Participants were excluded if they had: (a) deformities, fractures, trauma, or surgeries of the trunk and limbs; (b) conditions such as pregnancy, lactation, menstruation, or chronic dysmenorrhea; (c) severe cardiovascular, cerebral, hepatic, renal, or psychiatric disorders; (d) cerebral, otogenic, ophthalmic, or other diseases.

Healthy individuals were selected based on the absence of dizziness symptoms and related conditions, with demographic characteristics matched to those of the cervicogenic dizziness patients.The study received approval from the Ethics Committee of Guangzhou Panyu District Central Hospital (PYRC-2021–077), and informed consent was obtained from all participants prior to testing. Anthropometric parameters, including age, height, weight, and body mass index (BMI), showed no significant differences between the two groups (P > 0.05). See Table 1 for details.

**TABLE 1:** Demographic and anthropometric data of the sample (mean ± SD).

Group	Sex (male/female,n)	Age (years)	Body weight (kg)	Body height (m)	BMI (kg/m^2^)
Dizziness group (n = 20)	5/15	57.55 ± 6.33	58.35 ± 5.93	1.61 ± 0.08	22.59 ± 2.78
Health group (n = 20)	8/12	56.00 ± 4.72	62.55 ± 9.05	1.64 ± 0.07	21.31 ± 2.22
*χ* ^*2*^ */t*	1.026	0.878	−1.736	−1.386	1.616
*P*	0.311	0.386	0.091	0.174	0.114

### 2.2 Test program

#### 2.2.1 Testing instruments and requirements

The study utilized the Model 252 Prokin Balance Instrument (TECNOBODY, Italy) for postural assessment. This instrument has been validated in previous studies as a highly reliable and efficient tool for evaluating postural control (Dumanlidağ and Milanlioğlu, 2021).

Previous studies have demonstrated that the Model 252 Prokin Balance is capable of detecting subtle nuances or impairments that traditional clinical scales might overlook (Lin et al., 2020). This capability effectively mitigates the ceiling effect commonly associated with scale-based assessments. This distinctive attribute was a crucial factor in our selection of the Prokin Balance as the testing instrument for this study.

Prior to the assessment, the testing environment was maintained in a quiet state. The researcher provided the participants with detailed explanations regarding the purpose, procedures, and precautions of the assessment to ensure the process proceeded smoothly.

To minimize potential external influences such as environmental conditions and clothing, participants were instructed to wear loose-fitting attire and perform one or two pre-test exercises. Additionally, a quiet testing environment was maintained. These measures were implemented to ensure the accuracy and reliability of the data collected.

**FIGURE 1:**
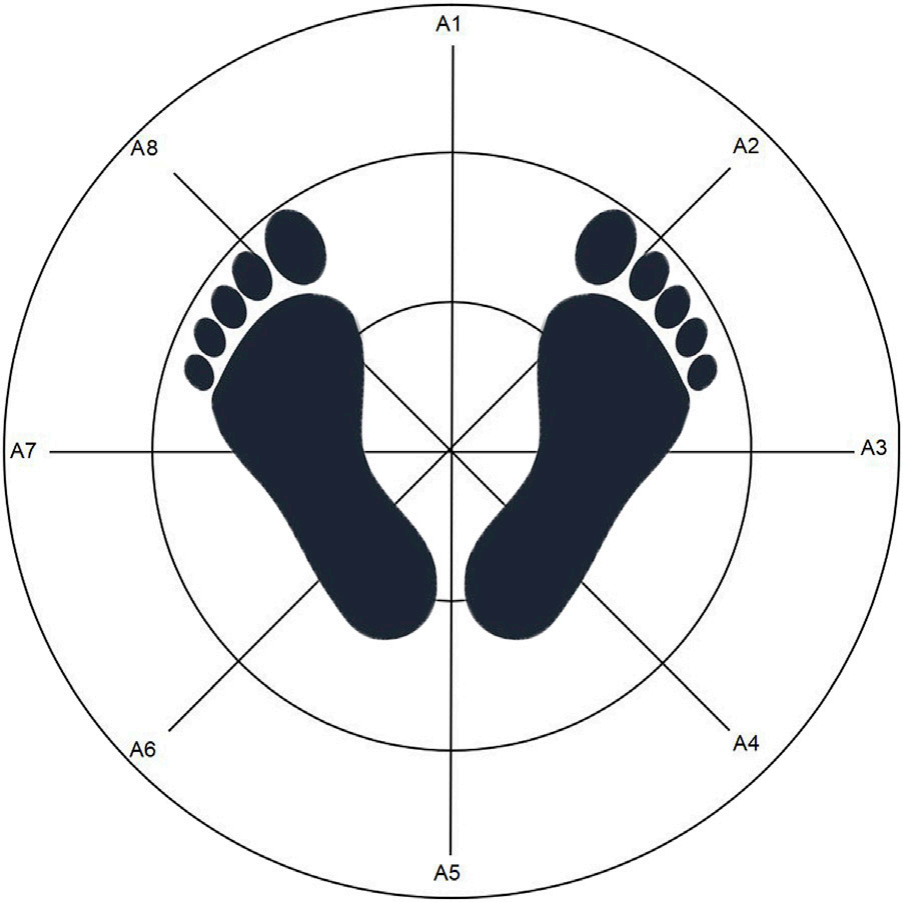
Schematic diagram illustrating the top-down view of a subject in the standard standing position.

Participants were instructed to adopt a standard standing position for the posture assessment. This involved standing barefoot on the pressure platform of the balance instrument, with feet apart and symmetrically aligned along the center axis (A1-A5). The feet were positioned together, with the second toe of the left foot pointing to A8 and the second toe of the right foot pointing to A2. The heels were aligned along the same transverse axis, and the inner ankles crossed the A3-A7 transverse axes. Participants were required to place their hands naturally at their sides and focus their gaze straight ahead on a 1-m achromatic target. See Figure 1 for reference.

#### 2.2.2 Test composition

**FIGURE 2:**
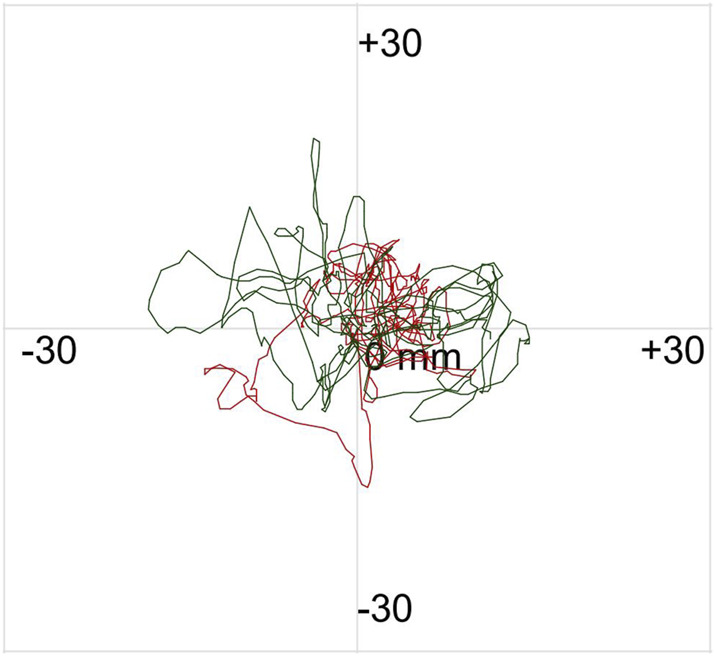
Statokinesiogram of the subject during the static balance test. The red and green lines depict the trajectory of the center of pressure swing with eyes open and closed, respectively.

The postural control assessment comprised a static balance test and a dynamic balance test (limits of stability). The static balance test included two visual input conditions: eyes-open and eyes-closed, each lasting 30 s (Zhang et al., 2020; Paillard and Noé, 2015). Participants were instructed to remain as still as possible during the test. Repeat each test twice. Figure 2 displays the statokinesiogram during the static balance test.

**FIGURE 3:**
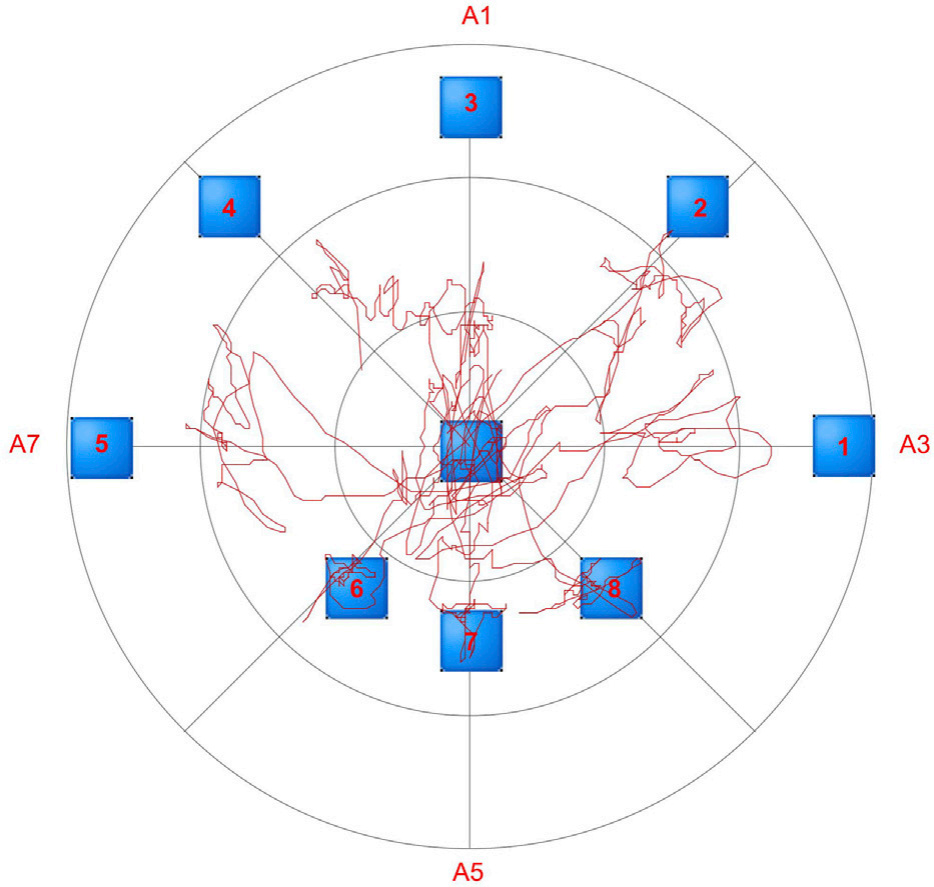
Motion trajectory diagram of the subject during the limits of stability test. The red line illustrates the movement trajectory of the human body’s center of pressure during limits of stability testing.

For the Limits of Stability Test, participants were tasked with moving a cursor as quickly and accurately as possible from the center of a computer screen to one of eight targets, arranged at 45-degree intervals around the center and highlighted sequentially. Participants were instructed to keep their feet stationary, avoid falling, and refrain from touching the bar during the test. They were also required to return the cursor to the center before the next target appeared. The test concluded once all eight targets had been displayed. This test was also repeated twice. Figure 3 illustrates the motion trajectory diagram of the subject during the limits of stability test.

#### 2.2.3 Observation indicators

Drawing upon insights from prior research (Sarabon et al., 2013; Hébert-Losier and Murray, 2020; Thomsen et al., 2017), we identified the following highly reliable and sensitive parameters as the primary observables for this study:

(a) Static balance: During the static balance test, the following parameters were recorded: average center of pressure X, average center of pressure Y, standard deviation of anteroposterior sway, standard deviation of mediolateral sway, average speed of anteroposterior sway (mm/s), average speed of mediolateral sway (mm/s), length of body sway (mm), and area of body sway (mm^2^). Larger values indicate poorer static balance stability (Walia et al., 2021).
(b) Romberg’s Ratio: This ratio primarily reflects the patient’s reliance on vision for postural control. In this study, we calculated the Romberg’s ratio using both the area and length of body sway, defined as the ratio of eyes-closed to eyes-open. A larger ratio signifies a higher degree of visual dependence (Dunn et al., 2024; Anagnostou et al., 2024).
(c) Limits of Stability: This was assessed by recording the total percentage of completion after the participant achieved the eight targets. A higher percentage indicates stronger limits of stability and a reduced risk of falls (Tomita et al., 2024).

#### 2.2.4 Statistical analysis

Statistical analysis was conducted using SPSS 21.0 software. Data conforming to a normal distribution were expressed as mean ± SD and analyzed using paired t-tests within groups and independent samples t-tests between groups. Data not conforming to a normal distribution were expressed as median and Interquartile range (IQR), with the Wilcoxon signed-rank test used for two related samples within groups, and the Mann-Whitney U test used between groups. Effect size (r) was used to calculate the power of the nonparametric tests. The significance level was set at α = 0.05.

## 3 Results

### 3.1 Comparison of static balance results between the two groups

In the dizziness group, all static balance indices were significantly higher when participants' eyes were closed compared to when their eyes were open (P < 0.05). Similarly, in the healthy group, static balance indices were also significantly elevated in the eyes-closed condition compared to the eyes-open condition, with the exception of the standard deviation of mediolateral sway (P > 0.05). Refer to Table 2 for detailed comparisons.

**TABLE 2:** Comparison of intra-group static balance test results for two groups of subjects with eyes open and closed within each group (Median (IQR)).

Group	Indicators		EO	EC	*Z-*value	*P-*value	Effect size (*r-value*)
Dizziness group	Standard Deviation of Sway	AP	4.0 (4.0,5.0)	5.0 (4.0,6.0)	−2.731	0.006**	−0.611
		ML	2.0 (2.0,3.0)	4.0 (3.0,5.0)	−3.808	0.000***	−0.851
	Average Speed of Sway (mm/s)	AP	6.0 (5.0,7.0)	9.5 (7.3,14.8)	−3.592	0.000***	−0.803
		ML	4.0 (3.0,4.8)	4.5 (3.3,6.0)	−2.068	0.039*	−0.462
	Area of Body Sway (mm^2^)		136.0 (110.5,202.0)	341.0 (290.0.445.8)	−3.808	0.000***	−0.851
	Length of Body Sway (mm)		240.0 (206.3,296.8)	354.0 (273.5,519.8)	−3.472	0.001**	−0.776
Health group	Standard Deviation of Sway	AP	2.0 (2.0,2.0)	3.0 (2.3,3.0)	−3.116	0.002**	−0.697
		ML	1.0 (1.0,2.0)	2.0 (1.0,2.0)	−1.508	0.132	−0.337
	Average Speed of Sway (mm/s)	AP	5.0 (4.3,6.0)	7.0 (6.0,9.0)	−3.584	0.000***	−0.801
		ML	3.0 (3.0,4.0)	4.0 (3.0,6.0)	−2.918	0.004**	−0.652
	Area of Body Sway (mm^2^)		62.5 (37.5,95.0)	93.5 (68.5,151.0)	−2.744	0.006**	−0.614
	Length of Body Sway (mm)		230.5 (185.3,266.5)	288.5 (236.8,339.5)	−3.043	0.002**	−0.680

AP: anteroposterior; ML: mediolateral; EO: Eyes-open; EC: Eyes-closed.

^*^P < 0.05,^**^P < 0.01,^***^P < 0.001.

Under the eyes-open condition, except for the average speed of mediolateral sway and the length of body sway (P > 0.05), all other static balance indices in the dizziness group were significantly higher than those in the healthy group (P < 0.05). In the eyes-closed condition, with the exception of the average speed of mediolateral sway (P > 0.05), the indices in the dizziness group were also significantly higher than those in the healthy group (P < 0.05). Refer to Table 3 for detailed comparisons.

**TABLE 3:** Comparison of inter-group static balance test results for two groups of subjects with eyes open or closed (Median (IQR)).

Group	Indicators		Dizziness group	Health group	*Z-*value	*P-*value	Effect size (*r-value*)
EO	Standard Deviation of Sway	AP	4.0 (4.0,5.0)	2.0 (2.0,2.0)	−4.669	0.000***	−0.738
		ML	2.0 (2.0,3.0)	1.0 (1.0,2.0)	−2.739	0.006**	−0.433
	Average Speed of Sway (mm/s)	AP	6.0 (5.0,7.0)	5.0 (4.3,6.0)	−2.077	0.038*	−0.328
		ML	4.0 (3.0,4.8)	3.0 (3.0,4.0)	−1.058	0.290	−0.167
	Area of Body Sway (mm^2^)		136.0 (110.5,202.0)	62.5 (37.5,95.0)	−4.328	0.000***	−0.684
	Length of Body Sway (mm)		240.0 (206.3,296.8)	230.5 (185.3,266.5)	−1.042	0.298	−0.165
EC	Standard Deviation of Sway	AP	5.0 (4.0,6.0)	3.0 (2.3,3.0)	−4.669	0.000***	−0.738
		ML	4.0 (3.0,5.0)	2.0 (1.0,2.0)	−4.601	0.000***	−0.727
	Average Speed of Sway (mm/s)	AP	9.5 (7.3,14.8)	7.0 (6.0,9.0)	−2.670	0.008**	−0.422
		ML	4.5 (3.3,6.0)	4.0 (3.0,6.0)	−0.822	0.411	−0.130
	Area of Body Sway (mm^2^)		341.0 (290.0.445.8)	93.5 (68.5,151.0)	−5.356	0.000***	−0.847
	Length of Body Sway (mm)		354.0 (273.5,519.8)	288.5 (236.8,339.5)	−2.178	0.029*	−0.344

AP: anteroposterior; ML: mediolateral; EO: Eyes-open; EC: Eyes-closed.

^*^P < 0.05,^**^P < 0.01,^***^P < 0.001.

### 3.2 Comparison of scatterplot results for the average center of plantar pressure between the two groups

**FIGURE 4:**
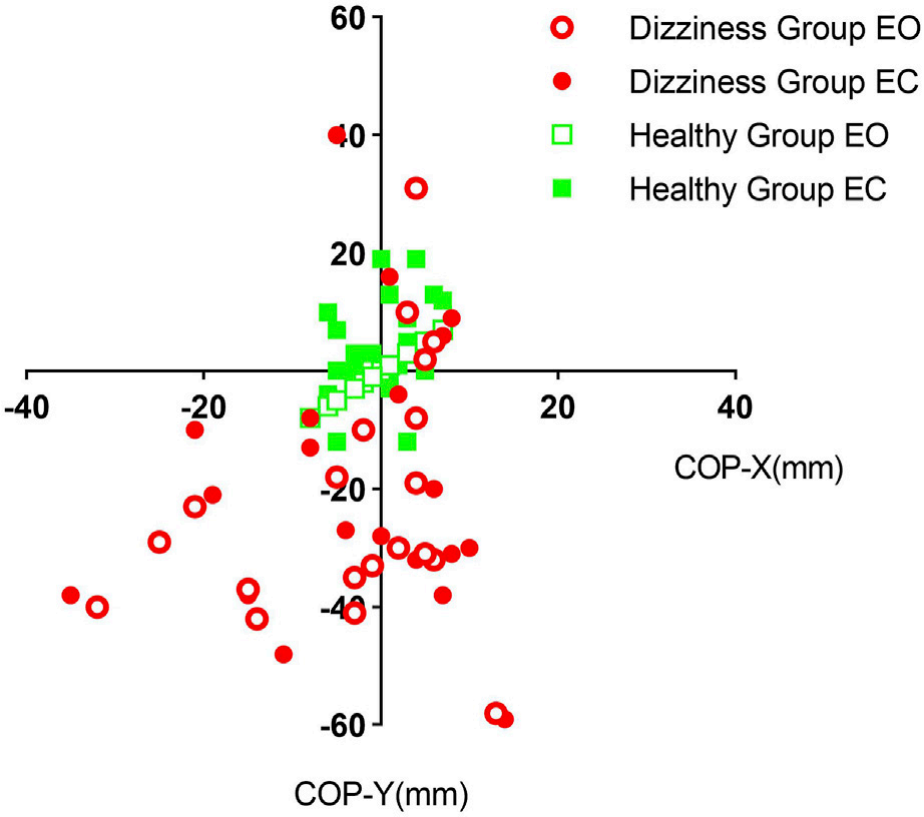
Scatter plots depicting the average pressure center distribution on the soles of the feet for the two groups of subjects with their eyes open and closed, respectively. EO: Eyes-open; EC: Eyes-closed; COP-X: average center of pressure X; COP-Y: average center of pressure Y.

Whether the eyes were open or closed, the average displacement of the center of foot pressure in the dizziness group exhibited a wide dispersion, deviating significantly from the central axis. In contrast, the distribution in the healthy group was more concentrated around the central axis. For further details, please refer to Figure 4.

### 3.3 Comparison of limits of stability and Romberg’s ratio between the two groups

**FIGURE 5:**
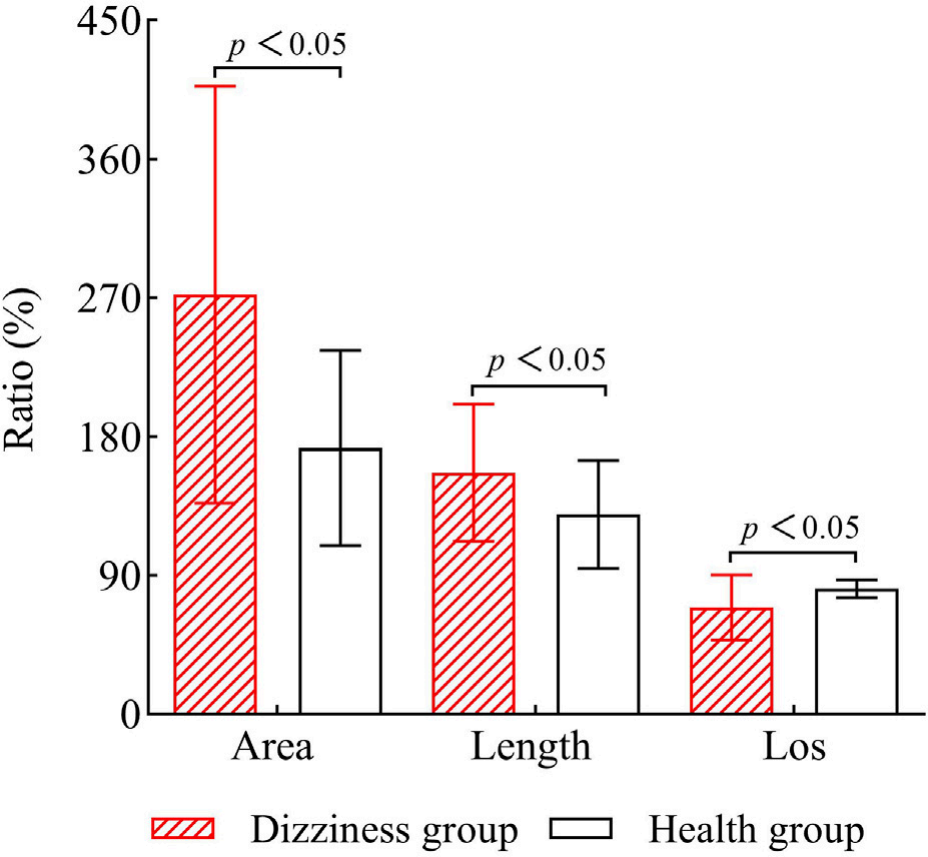
Comparison of Romberg’s ratio and limits of stability (LOS) between the two groups. LOS: Limits of Stability.

When compared to the healthy group, the dizziness group showed significantly greater area and length ratios (P < 0.05). Conversely, the percentage values for limits of stability were significantly lower in the dizziness group than in the healthy group (P < 0.05). See Figure 5 for detailed results.

## 4 Discussion

In this study, we conducted an in-depth analysis of the postural control characteristics in patients with cervicogenic dizziness using the highly reliable and efficient Prokin balance instrument. Our findings robustly support the hypothesis that static balance index values are higher in the eyes-closed condition compared to the eyes-open condition, both in healthy individuals and in patients with cervicogenic dizziness. Notably, the disparity in balance index values between these conditions, as well as the Romberg ratios, were significantly elevated in patients with cervicogenic dizziness compared to healthy participants. This indicates that individuals with cervicogenic dizziness exhibit poorer balance maintenance and a greater reliance on visual input when visual cues are removed, relative to their healthy counterparts. Additionally, the study revealed that patients with cervicogenic dizziness have a markedly reduced range of limits of stability and a significantly larger range of oscillations in the center of plantar pressure than the healthy population. These findings suggest that cervicogenic dizziness patients experience a restricted range of safe mobility during daily activities, diminished physical dexterity and adaptability, and a significantly heightened risk of falls. These findings provide crucial evidence for the development of targeted balance training programs. Leveraging this evidence, more effective training regimens can be designed to enhance patients' balance abilities and effectively prevent falls.

Cervicogenic dizziness, a condition resulting from functional or organic changes in neck structures, is primarily characterized by dizziness and impaired postural control (Micarelli et al., 2020). Epidemiological studies indicate that annually, approximately 15%–35% of patients seeking medical care report dizziness, a condition particularly prevalent among middle-aged and elderly populations (Koukoulithras et al., 2022). The increasing use of electronic devices has contributed to a rise in the incidence of cervicogenic dizziness within these age groups (Takahashi, 2018), which in turn exacerbates the global prevalence of fall incidents, imposing a substantial burden on patients' quality of life, physical and mental health, and socio-economic systems. Consequently, it is crucial to explore preventive strategies for falls in middle-aged and elderly patients with cervicogenic dizziness. A fundamental step is to thoroughly understand the balance and postural control characteristics in these patients, thereby guiding researchers in developing effective interventions, which hold significant implications for the diagnosis, treatment, and prevention of cervicogenic dizziness.

While previous studies have primarily investigated the balance function in patients with cervicogenic dizziness using methods such as the Balance Scale, these studies are limited in several ways. The balance parameters observed are relatively narrow and lack highly sensitive indices. Additionally, the procedures are cumbersome and exhibit deficiencies in accuracy and objectivity (Yahia, et al., 2009; Micarelli, et al., 2020). Consequently, these studies struggle to accurately, objectively, and comprehensively capture the characteristics of postural control disorders in patients with cervicogenic dizziness. Previous studies have investigated various parameters for assessing balance posture. For instance, the elliptical area is frequently employed to quantify balance posture performance, as it encompasses 90% or 95% of the total area in both the anterior-posterior and lateral directions, serving as a reliable indicator of overall posture performance. Typically, a smaller elliptical area signifies better postural stability (Asseman et al., 2004). Additionally, path length is regarded as an effective outcome measurement parameter, with smaller values indicating greater postural stability (Donath et al., 2012). Swing speed reflects the efficiency of the postural control system and characterizes the net neuromuscular activity required to maintain balance, making it the most reliable measurement method in experimental settings (Duarte and Freitas, 2010). Swing amplitude is also a dependable parameter and has been extensively utilized to analyze postural deficits in patients with neuromotor disorders, such as cerebral palsy, particularly when examining left-right directional movements (Pavão et al., 2014). However, some studies have noted that parameters like frequency and capture time exhibit relatively low sensitivity, especially when less influenced by visual cues, and their performance is not as significant as the aforementioned indicators (Sarabon et al., 2013).

Advanced balance instrumentation provides a quantitative analysis of the body’s balance function under both dynamic and static conditions, uncovering issues that traditional observational methods and scale evaluations might miss. This allows for an accurate and objective assessment of a patient’s balance status (Haliloglu et al., 2019). This technology has been successfully applied to the assessment and training of balance functions in patients with diabetes and stroke, demonstrating its efficacy. (Reyhanıoglu et al., 2024; Lin et al., 2020). In this study, we utilized the Model 252 balancing instrument from Italy to evaluate the static balance and stabilization limits of middle-aged and elderly patients with cervicogenic dizziness. The balance instrument offers a comprehensive evaluation of balance issues from both static and dynamic perspectives and includes the highly reliable and sensitive balance indicator parameters previously mentioned. This study’s implementation will effectively address the constraints of prior research and conventional methodologies in evaluating postural disorders in individuals with cervicogenic dizziness.

Our findings demonstrate that both healthy individuals and patients with cervicogenic dizziness exhibit diminished static balance in the eyes-closed condition compared to the eyes-open condition, underscoring the moderating effect of visual input on static balance. Regardless of visual input, all static balance indices were significantly higher in patients with cervicogenic dizziness than in healthy subjects, indicating significant static balance dysfunction in these patients that is independent of visual input. Furthermore, the Romberg ratios for body sway length and area were significantly elevated in patients with cervicogenic dizziness compared to healthy subjects, suggesting a greater visual dependency in static balance regulation among the cervicogenic dizziness.

The Romberg ratio is a classic and well-established parameter in neurological assessments, though its sensitivity remains a topic of debate within academic circles, with scholars expressing differing views. Our research findings align with those of Putri et al., suggesting that the Romberg ratio can, to some extent, reflect a patient’s postural balance ability (Putri and Komalasari, 2025), despite differences in the patient populations studied. Additionally, Tjernström et al. questioned the reliability and validity of the Romberg ratio as an assessment tool for postural control in healthy young adults (Tjernström et al., 2015). We speculate that this discrepancy may be due to the influence of disease factors. The presence of disease may amplify changes in the Romberg ratio, a hypothesis that warrants further investigation in future studies.

Despite this increased visual reliance, static balance dysfunction persists in middle-aged and elderly patients with cervicogenic dizziness. This may be attributed to disturbances in neck proprioception, as balance control heavily depends on the integration of visual, vestibular, and proprioceptive inputs (Cao et al., 2021). When proprioception is compromised, the body compensates by relying more on other senses, yet increased visual dependence often falls short of fully compensating for proprioceptive deficits (Meier et al., 2016). This explains why patients with cervicogenic dizziness, despite their increased visual dependence, demonstrate inferior static balance capabilities compared to healthy individuals.

In the discussion of the pathogenesis of cervicogenic dizziness, although four main types were initially proposed, subsequent research has refuted the existence of neurosympathetic cervicogenic dizziness, and rotational vertebral artery cervicogenic dizziness is now considered extremely rare. Migraine-type cervicogenic dizziness still requires further investigation and validation. In contrast, proprioceptive cervicogenic dizziness has emerged as the most common and widely accepted mechanism (Li and Peng, 2015). This underscores the central role of proprioceptive dysfunction in the pathogenesis of cervicogenic dizziness and its significant impact on postural control disorders.

Proprioceptive information, transmitted by proprioceptors located in joints, muscles, and tendons, is crucial for perceiving body position and movement (Riemann and Lephart, 2002; Tuthill and Azim, 2018). In the cervical region, approximately 50% of proprioceptors are distributed in the C1-C3 joint capsules (Hulse, 1983), highlighting the significant role of the upper cervical spine in proprioceptive regulation (McLain, 1994; Pettorossi and Schieppati, 2014). The upper cervical spine (including the atlanto-occipital and atlanto-axial joints) is responsible for the majority of flexion and rotational movements of the neck (Li and Peng, 2015; Savitz and Caplan, 2005; Swartz et al., 2005), with its mobility dependent on the synergistic action of ligaments and muscles. Ligaments such as the transverse ligament primarily maintain the stability of the upper cervical spine (Steilen et al., 2014), while muscle groups like the suboccipital muscles not only regulate neck motion (Yamauchi et al., 2017) but also serve as sensory receptors due to their high density of muscle spindles, playing a crucial role in postural adjustment (Kulkarni et al., 2001). Therefore, cumulative abnormal stimuli from prolonged poor posture, as well as trauma and degenerative changes, may affect the structural function of the upper cervical spine, subsequently impairing proprioception, leading to dizziness and postural abnormalities (Chu et al., 2020; L'Heureux-Lebeau et al., 2014).

Based on the aforementioned anatomical and physiological evidence, as well as the positive effects of proprioceptive training on postural stability in patients with cervicogenic headaches and healthy elderly individuals (Martínez-Amat et al., 2013; Emam et al., 2024), the findings of this study may offer a new perspective for the rehabilitative treatment of cervicogenic dizziness. Proprioceptive training could be equally applicable to patients with cervicogenic dizziness, potentially enhancing their perception of head position and movement, thereby improving their postural control and reducing symptoms of dizziness.

It is important to note that cervical pain is not only a symptom of cervicogenic dizziness but may also be a contributing factor, with a close association between the two. Nearly half of patients with neck pain experience cervicogenic dizziness (Vural et al., 2021). Neck pain is a symptom with high specificity (100%) but low sensitivity (68%) for cervicogenic dizziness (L'Heureux-Lebeau et al., 2014). However, pain itself may negatively impact cervical proprioception. Pioneering studies have found that individuals with cervical spine pain exhibit impaired sensorimotor control (Treleaven, 2008), a phenomenon particularly pronounced in patients with chronic pain (Lee et al., 2008). These findings support the notion that pain may alter cervical spine proprioception and afferent signals, leading to sensory mismatch.

In this study, we observed that the average center of plantar pressure in patients with cervicogenic dizziness exhibited a widely dispersed distribution in both eyes-open and eyes-closed conditions, whereas healthy individuals showed concentrations near the axis. This finding indicates that patients with cervicogenic dizziness experience greater shifts in their center of gravity during static balance assessments, reflecting poorer balance control compared to healthy individuals. Furthermore, dynamic balance stability was also compromised in patients with cervicogenic dizziness, as evidenced by the Limits of Stability Test results. Middle-aged and elderly patients with cervicogenic dizziness demonstrated reduced limits of stability in all eight directions, coupled with an increased risk of falling compared to their healthy counterparts.

Notably, in the comparison between patients with cervicogenic dizziness and healthy individuals, we found that the disparities in the average speed of anteroposterior sway were more pronounced than those in the mediolateral direction. This pattern was consistent across both eyes-open and eyes-closed conditions. Similar findings have been reported in patients with low back pain and cervical spine disorders (Soliman et al., 2017; Field et al., 2008). This may be due to the involvement of the hip, knee, ankle, and lumbar spine joints during anterior-posterior movements in the sagittal plane, which allows for a greater range of motion. In contrast, lateral movements in the frontal plane primarily involve only the hip, ankle, and lumbar spine joints.

Alternatively, the simplicity of static balance tests may not be sufficient to reveal balance differences, as significant variations in balance function are often more apparent during complex movements (Promsri et al., 2020). The Limits of Stability Test represents such a complex task, suggesting that it may serve as a more precise assessment tool for detecting balance discrepancies.

## 5 Limitations

This study has several inherent limitations. Firstly, while it primarily focuses on analyzing the static and dynamic balance characteristics of patients with cervicogenic dizziness, it does not explore the specific causes and mechanisms underlying these balance characteristics, leaving room for speculation. Secondly, the study predominantly targets middle-aged and elderly populations, where prevalence is high, without distinguishing among other age groups or genders. This limitation restricts the generalizability of the findings across different ages and genders. Lastly, although cervicogenic dizziness remains a diagnosis of exclusion, the study by Treleaven et al. demonstrated that a positive response to the modified cervical torsion test and the head-neck differentiation test can significantly enhance the diagnostic accuracy for cervicogenic dizziness (Treleaven et al., 2020). While our study employed similar methods, the modified testing protocols proposed by Treleaven et al. exhibit superior scientific rigor and standardization. Existing research suggests that gender differences may influence balance function (Ozcan Kahraman et al., 2018), indicating that further studies with gender differentiation could more accurately elucidate the balance function characteristics in patients with cervicogenic dizziness.

## 6 Conclusion

In conclusion, middle-aged and elderly patients with cervicogenic dizziness exhibit marked impairments in postural control, as demonstrated by significant reductions in static balance and limits of stability, as well as increased and dispersed center of gravity sway. These factors collectively indicate a heightened risk of falling. Additionally, maintaining postural control appears to rely, to some extent, on visual compensatory mechanisms, indicating a degree of visual dependence that may relate to disruptions in neck proprioceptive function.

## Data Availability

The original contributions presented in the study are included in the article/supplementary material, further inquiries can be directed to the corresponding authors.
